# Position of the Society for Nutrition Education and Behavior: The Importance of Including Environmental Sustainability in Dietary Guidance

**DOI:** 10.1016/j.jneb.2018.07.006

**Published:** 2019-01

**Authors:** Donald Rose, Martin C. Heller, Christina A. Roberto

**Affiliations:** 1 School of Public Health and Tropical Medicine, Tulane University, New Orleans, LA; 2 Center for Sustainable Systems, School for Environment and Sustainability, University of Michigan, Ann Arbor, MI; 3 Perelman School of Medicine, University of Pennsylvania, Philadelphia, PA

**Keywords:** agriculture, climate change, dietary choice, dietary guidance, environment

## Abstract

It is the position of the Society for Nutrition Education and Behavior that environmental sustainability should be inherent in dietary guidance, whether working with individuals or groups about their dietary choices or in setting national dietary guidance. Improving the nutritional health of a population is a long-term goal that requires ensuring the long-term sustainability of the food system. Current environmental trends, including those related to climate change, biodiversity loss, land degradation, water shortages, and water pollution, threaten long-term food security and are caused in part by current diets and agricultural practices. Addressing these problems while producing more food for a growing population will require changes to current food systems. Dietary choices have a significant role in contributing to environmental impacts, which could be lessened by consuming fewer overconsumed animal products and more plant-based foods while reducing excess energy intake and the amount of food wasted. Discussion of sustainability within governmental dietary guidance is common in many countries, is consistent with previous US guidelines, and is within the scope of authorizing legislation. Dietary choices are a personal matter, but many American consumers are motivated by a concern for the environment and would welcome sound advice from credentialed nutrition professionals. More opportunities are needed for developing such interdisciplinary knowledge among nutritionists.

## Introduction

*It is the position of the Society for Nutrition Education and Behavior (SNEB) that environmental sustainability should be inherent in dietary guidance, whether working with individuals or groups about their dietary choices or in setting national dietary guidance. Improving the nutritional health of a population is a long-term goal that requires ensuring the long-term sustainability of the food system*. In early 2015, the Dietary Guidelines Advisory Committee^1^ (DGAC), a committee of experts charged with developing dietary guidance for the US population, released their scientific advisory report to the Secretaries of Agriculture and Health and Human Services. The DGAC devoted a section to the issue of food sustainability, defining a sustainable diet as “a pattern of eating that promotes health and well-being and provides food security for the present population while sustaining human and natural resources for future generations.”^1^ Based on existing evidence, the DGAC asserted that a diet higher in plant-based foods and lower in animal-based foods is healthier and associated with a lower environmental impact than the current U.S. diet, and that this could be achieved without excluding any food groups.^1^ However, the final *Dietary Guidelines for Americans* (DGA),^2^ produced by the US Department of Agriculture (USDA) and the Department of Health and Human Services, make no mention of this issue. This raises a series of questions the current authors attempt to address in this article: Should we, as a society, care about the environmental sustainability of the food system? Does what we eat have a significant impact on the environment? Is dietary guidance compatible with efforts to promote such sustainability? Why was the DGAC discussion on sustainability not included? In answering these questions, this article lays out the scientific rationale for the SNEB's position statement. After a brief description of current environmental problems, the article discusses the challenges faced in meeting future food needs and the recent science behind assessing the environmental impacts of foods and diets. A subsequent section discusses sustainability and dietary guidance in this country and others and some specific recommendations for dietary guidance and research. Throughout this article, the authors focus on the environmental dimension of sustainability rather than its health, social, or economic dimensions. It is vitally important to consider these other dimensions of sustainability but they are beyond the scope of this article.

## Current Environmental Problems

Global climate change is one of the most urgent problems that exists today. In their latest report, the Intergovernmental Panel on Climate Change,^3^ a United Nations scientific body tasked with interpreting the latest information on climate change, indicated that despite a growing number of attempts to deal with this problem, the growth of greenhouse gas emissions (GHGE) has accelerated over the past decade. The report was the starkest warning to date, indicating that shifts in food production areas, more frequent and intense heat waves and extreme precipitation events, warming and acidifying oceans with decreased fishery yields, and water scarcity all present risks to global food security and could threaten generations of gains in fighting poverty and hunger if action is not taken. The overwhelming majority of scientists agree regarding the cause of climate change,^3^, ^4^, ^5^, ^6^ although some scientists do not.^7^ Many of the previous predictions by climate scientists have already been borne out.^3^, ^6^ The planet is warming: 15 of the 16 warmest years on record have occurred during the 21st century.^8^ The arctic ice sheets are melting, sea levels are rising, and there are more extreme weather events.^3^ The urgency of the situation motivated the Paris Accords of 2015, a historic agreement between 195 countries to limit GHGE.^9^

In addition to global climate change, pressing environmental problems include loss of biodiversity, land degradation, fresh water shortages, and water pollution. The importance of biodiversity for the functioning of ecosystems and human well-being has been officially acknowledged since the creation of the Convention on Biological Diversity at the Earth Summit in Rio de Janeiro, Brazil, in 1992.^10^ Biodiversity, which reflects the number and variety of living organisms, boosts the productivity, resilience, and sustainability of ecosystems, which in turn offers many benefits to humans, such as soil formation and retention, pollination of plants, regulation of climate, as well as provisioning of resources for foods and pharmaceuticals.^11^ Unfortunately, despite conservation efforts by some of the signatories to this multilateral treaty, biodiversity loss at a global scale is continuing and even accelerating in some cases.^12^, ^13^ This biodiversity loss is likely to be human-induced and is resulting in a mass extinction, the first since the Cretaceous, when the dinosaurs became extinct.^13^ Some authors^14^, ^15^ demonstrated that the impacts of biodiversity loss on ecosystem functioning may rival the impacts of other drivers of change, such as global climate change.

Reduction in the productive capacity of land, or land degradation, owing to loss of soil quality, biodiversity loss, salinization, or water depletion, is another serious problem. The United Nations Food and Agriculture Organization conducted a global assessment of the state of the planet's land resources and found that about 33% of all land is moderately or highly degraded.^16^ Main contributors to this degradation are crop and soil management techniques, deforestation, and overexploitation (eg, overgrazing).

Changes in water resources are also a concern in terms of both quantity and quality. A global analysis of the freshwater systems that provide water for humans and are important for ecosystem functioning (as assessed by biodiversity) found that 80% of the world's population lives where water security for humans or for ecosystem functioning is highly threatened.^17^ Water quality is affected by runoff of fertilizer and manure from agricultural soils and nutrient- and toxic chemical–containing effluents from industry. Over-enrichment of water by the nutrients nitrogen and phosphorus can result in algal blooms, depletion of oxygen, and destruction of aquatic life. Referred to as eutrophication, over three quarters of the assessed US continental coastal area, and many inland water bodies as well, are experiencing moderate to high levels of this problem.^18^, ^19^

A serious concern is that agriculture, including livestock production, is one of the largest contributors to environmental damage, including extensive clearing of forests, overuse of freshwater resources, widespread water pollution, and global climate change.^20^ Only 62% of the world's crop production is destined directly for human consumption; most of the rest is used for animal feed.^20^ The United Nations Food and Agriculture Organization estimates that GHGE associated with the production of meat and dairy products alone account for 14.5% of all GHGE globally,^21^ although in the US, livestock production accounts for 5.6% of GHGE owing to the greater impact of the transportation and energy sectors.^22^, ^23^ Overall, agriculture is responsible for 70% to 80% of all human water withdrawals globally^24^ and occupies about 38% of the earth's ice-free land.^20^ Environmental degradation can further diminish agriculture's productivity: it is predicted that up to 25% of world food production may be lost during the 21st century as a result of climate change, land degradation, water scarcity, and other causes.^25^ A global modeling study^26^ also showed the potential impacts of climate change on reduced food production and related deleterious impacts on human health.

## Meeting Our Future Food Needs

Currently an estimated 795 million people in the world lack adequate energy from food^27^ and over 2 billion people experience micronutrient malnutrition.^28^ At the same time, nearly 2 billion people in the world are overweight or obese.^29^ The high prevalence of overnutrition is a relatively recent global phenomenon which has resulted in part from increasing household incomes and westernization of the diet in middle- and even low-income countries.^30^, ^31^, ^32^ These problems are compounded by estimates that the world's population will grow by an additional 2.2 billion, totaling 9.7 billion, by 2050.^33^ Along with these trends, the demand for food is estimated to double by midcentury.^20^ This raises 2 interrelated challenges for the food, nutrition, and agricultural communities: (1) How do we meet the food and nutritional needs for this increased population, in addition to those whose needs are currently not met? (2) How do we accomplish this while also addressing these major environmental problems, which threaten to decrease the earth's carrying capacity?

A number of strategies have been suggested to meet these 2 long-term food challenges.^20^, ^32^ Two such strategies that come up repeatedly are changes in consumer demand, and sustainable intensification, or achieving more food production from existing farmland with far less environmental pressure.^34^ Intensification in the US dairy industry, for example, has come with less GHGE per unit of production. In 2007, the carbon footprint per billion kilograms of milk produced was only 37% of what it was in 1944.^35^ Another study found a reduction in the resource use and the carbon footprint of US beef production since the late 1970s.^36^

Nevertheless, repeated projection studies demonstrated that closing global yield gaps and other sustainable intensification measures will be insufficient to prevent further agricultural expansion and achieve the deep emission cuts needed to meet the Paris climate commitment; demand-side reductions will be necessary.^37^, ^38^, ^39^ Such demand-side reduction strategies typically take the form of reducing, although not necessarily eliminating, the amount of animal products in the diet, particularly in high-income countries.^20^, ^32^^,^^40^, ^41^, ^42^ The next 2 sections review the scientific basis for this strategy.

## Life-Cycle Assessment and the Environmental Impact of Foods

Nutritionists may recognize the term *life cycle* in the context of studying nutritional needs and how they change during the course of human life. Other scientists, such as industrial ecologists or environmental engineers, also use the term *life cycle* but in reference to products rather than humans. Life-cycle assessment (LCA) was developed as an analytical framework to assess the potential environmental impacts of industrial production systems across each stage of a product's life cycle, from resource extraction (cradle), manufacturing, and distribution to use and end-of-life disposal (grave).^43^ An LCA offers a means of considering, for example, how the energy and emissions associated with building a car compare with those from operating a car over its lifetime and disposing of it at end of life. This allows abatement strategies to focus on where they will have the greatest consequence. An LCA can also ensure that efforts to reduce environmental impacts at 1 stage (say, manufacturing) do not simply shift impacts to a different stage (say, disposal).

Ideally, LCA includes a broad range of environmental impact categories (eg, GHGE, nonrenewable energy use, eutrophication, acidification, ozone depletion, water use, land use) so that trade-offs between impact categories can be evaluated and potential shifts from 1 impact category to another can be detected when comparing scenarios.^43^ However, data availability, the scope of a given study, and the uncertainty introduced with some impact assessment methods often limit the impact categories that are practical or desirable.^43^ Carbon footprinting typically refers to a unique LCA approach that focuses only on GHGE over a product's life cycle.^43^

Over the past 2 decades, the LCA framework has been applied to food and agricultural systems, addressing many of the unique challenges that arise when considering these complex systems.^44^, ^45^ Early food LCAs focused on questions related to packaging: for example, comparing single-use milk cartons with disposable and refillable bottles. In the early 1990s, LCAs began to emerge that incorporated food production.^44^ Since then, hundreds of studies have been conducted on individual foods, some focused on agricultural production and others covering processing, distribution, consumption, and disposal aspects.^46^, ^47^, ^48^, ^49^ The field continues to grow and develop and is hosting its 11th international conference on the topic of LCA of food.^50^

In general, results from food LCA studies show that environmental impacts from food production are higher for animal products and higher still for ruminant animals. For example, the Figure displays the GHGE from the production of different foods in terms of their global warming potential, or carbon dioxide-equivalents (CO_2_eq), from a review of studies throughout the world. This unit of measure is used to place carbon dioxide, methane, nitrous oxide, and other gases on the same scale based on their relative contributions to global climate change. For vegetables and fruits, values are typically <1 kg CO_2_eq/kg of commodity. At the other end of the spectrum, beef has a value >30. Beef and products from other ruminant animals have such high values in large part because of methane gas released during enteric fermentation (part of ruminant digestion) and manure management, but also because of the energy used in growing feed grains and nitrous oxide released from soils receiving nitrogen fertilizers.^51^ Methane and nitrous oxide have 28 and 265 times the global warming potential of carbon dioxide, respectively, which makes relatively small emission quantities of these gases impactful.^52^

There are important caveats to keep in mind regarding the Figure. First, animal-based protein foods are particularly important in undernourished populations.^40^ Second, use of organic wastes by animals on the farm or from the food processing industry reduces the impact of livestock production.^53^ Third, manure digesters on livestock farms can turn the propensity of manure to generate methane into a resource; such biogas recovery projects are developing throughout the US.^54^ Fourth, although the data in this figure are based on averages from a wide range of studies, there is sizable local variation in impacts of specific foods owing to differences in production techniques, soil conditions, seasonality, and other factors. Fifth, weight is a convenient, but imperfect basis for comparison of different foods. Other bases, such as caloric or nutrient content, can also be used, depending on the purpose. Finally, the Figure does not consider other environmental impacts such as water use, which might show a different ranking of impacts across food types.

**Figure:**
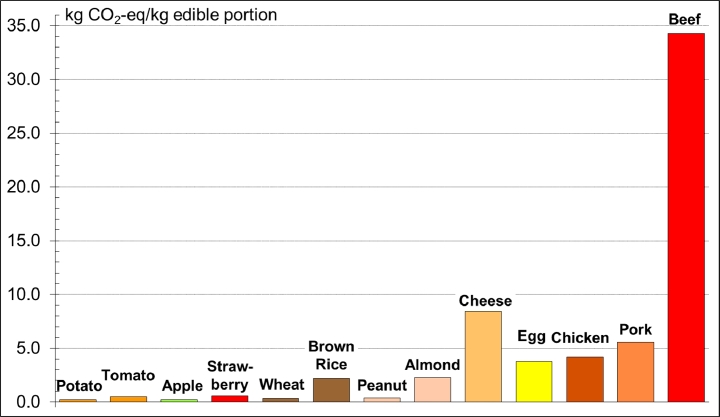
Greenhouse gas emissions for the production of selected foods (kilograms of CO_2_-eq per kilogram of edible portion of commodity). CO_2_-eq indicates carbon dioxide equivalents, which puts methane, nitrous oxide, and carbon dioxide, all global warming gases, on the same scale. Edible portion refers to the part of the commodity that can be eaten: in other words, without the bone on the beef, without the shell on the peanut, without the core or stem on the apple, etc. Data are from Heller et al.^60^

## Environmental Impact of Total Diets

A number of authors combined results from individual LCA studies to develop databases of environmental impacts. Much of this work was motivated by the goal of evaluating complete diets. For example, a database of the GHGE from food was developed for use in the United Kingdom.^55^ The environmental impacts of diets were also studied in France,^56^ Germany,^57^ and the Nordic countries.^58^ In the US, several teams compiled food LCA studies to evaluate complete diets.^59^, ^60^, ^61^

### Impacts Throughout the Food System

As mentioned, LCA studies on food can include the production, processing, distribution, consumption, and disposal phases of a food product. Some studies of complete diets compared the relative environmental impacts of these different phases or specific processes within a phase, which are discussed subsequently.

#### Production-level impacts

An important issue at the production level is the relative environmental impacts from differing methods of production. A comparison of organic vs conventional production exemplifies this. Recent reviews of the literature drawing from international production contexts indicated that the results are mixed, varying by the specific impact under consideration.^58^, ^62^^,^^63^ For example, organic agriculture has clear benefits regarding local biodiversity, soil quality, and reduced ecotoxicity (the toxic effects of pollutants on biological organisms) from pesticide use.^58^, ^62^^,^^63^ However, because yields are often higher with conventional agriculture,^58^, ^62^ the amount of land used is typically lower for conventional than for organic agriculture.^58^ As for global climate change, the results are mixed based on the crop. A recent review found that for the grains studied, organic agriculture had a lesser impact on global climate change than did conventional agriculture but the reverse was true for poultry, eggs, beef, and some produce items such as carrots and tomatoes.^58^ Specific differences between organic and conventional agriculture depend not just on the crop under investigation but also on the details of the production systems being compared.^62^, ^63^ Agricultural systems are complex, as are their environmental impacts, so increasing organic agriculture may benefit local biodiversity, soil quality, and ecotoxicity levels, but this would not always translate into reduced impacts on global climate change or land use.^58^, ^62^^,^^63^

#### Transportation and diet composition impacts

Several authors considered the relative environmental impacts resulting from the transportation of food vs the choice of specific foods that compose an entire diet.^58^, ^64^^,^^65^ In comparing the current average Danish diet and a new Nordic diet that was developed to be healthy and environmentally sustainable,^66^ researchers found that the greatest overall benefit of the new Nordic diet came from the differences in diet composition (ie, less meat and more plant foods), whereas transportation (ie, reduced long-distance transport of imported commodities) reduced impact to a lesser degree.^58^ Modifications of the average diet in the United Kingdom showed that reducing meat consumption, particularly of ruminant animals, had a much greater impact on lowering GHGE than did reducing the imports of produce from air shipping.^64^ In a US study of the transportation of food, the production phase was much more important; it contributed 83% of the food carbon footprint, whereas transportation represented only 11%.^65^ Given their results, the authors suggested that a dietary shift away from red meat and dairy, even a modest one, could accomplish a greater reduction in GHGE than buying all foods locally.^65^

Other studies focused solely on food choices. In the US, another modeling study found that replacing beef with plant-based alternatives could have substantial positive impacts on the environment, including a significant reduction in GHGE and land use, while offering an improved intake of many important nutrients.^67^ Among Seventh Day Adventists in California, estimates of the resources (including energy, pesticides, and land) needed to produce food for the diets of nonvegetarians were greater than for vegetarians; the differences resulted primarily from the inclusion of beef.^68^ Similarly, the diets of vegans and vegetarians were associated with lower GHGE compared with fish eaters and meat eaters in the United Kingdom.^69^

Studies that consider both diet composition and transportation effects showed that diet composition has a much greater influence on environmental impacts.^58^, ^64^^,^^65^ These studies, as well as those that focus solely on diet composition, were typically modeling studies based on assumptions and data inputs from a particular country. However, they consistently showed that diets with fewer animal products, particularly ruminants, have a lower environmental impact.^58^, ^64^^,^^65^^,^^67^, ^68^, ^69^

#### Food loss impacts

Another major contributor to negative environmental impacts is food loss, the edible amount of harvested food that is not consumed. In the US, this is largely made up of food waste, food that is produced but not eaten because of human behaviors (such as plate waste by consumers or discards by consumers or retailers because of appearance or expired sell-by dates), as opposed to losses owing to spoilage from mold or pests.^59^ Food loss is a big problem in the US and around the world because of its sheer magnitude and overall environmental impact.^70^, ^71^ Not only does lost food have all of the environmental impacts of food that is consumed, it has additional impacts from the disposal process with none of the benefit for human nutrition.^59^ Food losses appear to be an increasing problem in the US. One study analyzed the difference between the energy value of the US food supply and the predicted energy intake of the population based on sophisticated metabolic equations relating intake to body weight.^72^ It found a 50% increase in food losses in the 3 decades beginning in 1974, reaching more than 1400 kcal/person per day by 2003. The impact of such losses is substantial. Another study found that food losses contributed about 28% of the carbon footprint of the average US diet.^59^

### Impacts of Diets That Meet Government Recommendations

Another important line of research investigated the potential impact on the environment of diets that follow national dietary recommendations. In the Netherlands, the official recommended diet was compared with the current Dutch diet, using a sustainability score based on GHGE and land use.^73^ The recommended diet scored significantly better on sustainability, as did a number of other diet patterns examined, including Mediterranean, vegetarian, and vegan diets. Another study analyzed the GHGE from the average diet in the United Kingdom and then used linear programming optimization techniques to develop a diet that would meet that country's nutritional recommendations while minimizing GHGE.^74^ The researchers found that such a diet could result in a 36% decrease in emissions from the baseline diet.

A number of studies were conducted in other countries that either compared average diets with a recommended diet or developed a new diet through optimization techniques to lower GHGE while meeting dietary recommendations. In France, Spain, Sweden, Germany, Denmark, and New Zealand, those studies all showed that diets that met recommendations could lower GHGE.^57^^,^^75^, ^76^, ^77^ A study of all countries in the European Union found that shifting current consumption to diets that meet generally accepted dietary recommendations and have reduced meat consumption could lead to an 8% reduction in the environmental impacts of food.^78^

In the US, 4 studies examined the impacts of diets that meet the 2010 DGA. If the USDA food plan were to be consumed at recommended energy intakes, 1 study estimated that there would be a reduction in GHGE of about 1%.^59^ Adoption of the DGA's vegetarian plan would result in a further reduction in GHGE of 33%, or 53% for the vegan plan.^59^ Other analysts repeated this exercise for the US but took a different approach to estimating current *per capita* caloric intake and concluded that a shift to a recommended food mixture and caloric intake would increase GHGE by 6% and increase the energy and water used by the food system by 38% and 10%, respectively.^61^ Unlike the first study mentioned previously, this second study did not estimate impacts from a shift to the vegetarian or vegan food patterns listed in the 2010 DGA. A third study^79^ examined the number of persons who could be fed per unit of US agricultural land (called the carrying capacity) and found that a diet shift toward plant-based diets had the significant potential to increase this carrying capacity, with a lactovegetarian diet scoring the highest. The study emphasized that an optimal carrying capacity includes some livestock that can take advantage of western rangelands and other marginal lands, but this optimum would require significant decreases in animal-based foods from the current US diet. A fourth study^80^ examined a number of environmental impacts, including climate change, and land, water, and energy use, of the DGA's recommended food pattern and its vegetarian and vegan adaptations. Overall, the vegan diet had the lowest impacts, and the authors concluded that the environmental impact of a diet is mainly related to the consumption of animal products.

These studies, as well as others that analyzed different dietary scenarios, highlight the importance that changes in diet composition could have in reducing environmental impacts. A systematic review of 14 recent peer-reviewed diet scenario studies conducted mostly in European countries concluded that changing current diets could have a significant impact on the environment, potentially reducing GHGE and land use from diet by up to 50%.^81^ The largest potential changes were seen in diets that reduced the amount of meat.

## Previous Incorporation of Sustainability in Dietary Guidelines

Given the research showing that diets that follow national dietary guidelines for a healthy diet could lower environmental impacts,^57^^,^^75^, ^76^, ^77^ it is unsurprising that a number of dietary guidelines panels throughout the world have considered the sustainability of the food system when producing recommendations.^82^ This has been the case in Australia, Brazil, Denmark, Estonia, Finland, Germany, the Netherlands, Qatar, Sweden, the United Kingdom, and Uruguay (Table).^58^^,^^82^, ^83^, ^84^, ^85^, ^86^, ^87^, ^88^, ^89^, ^90^, ^91^, ^92^, ^93^, ^94^, ^95^, ^96^ The information considered and the recommendations made regarding sustainability vary among these countries. Some have suggested that consumers eat more food in season, eat fish from sustainable sources, eat more plant foods, reduce food waste, or consume only enough food to maintain energy balance. A number of countries have recommended consuming less meat or eating less animal food.

**Table:** Dietary Guidance Recommendations Addressing Sustainability in Various Countries

Recommendation	Australia^83^	Brazil^84^	Denmark^58^, ^85^	Estonia^82^, ^86^	Finland^87^, ^88^	Germany^89^, ^90^	The Netherlands^91^	Qatar^92^	Sweden^93^	United Kingdom^94^, ^95^	Uruguay^96^
Eat more sustainable food	X			X	X					X	
Consume minimally processed/nutrient-dense foods	X	X						X			
Eat more plant foods		X		X	X	X	X	X	X	X	X
Eat fewer animal foods		X					X				
Eat less meat			X		X	X			X	X	
Meal plan/store food for later use	X								X		
Consume only enough calories for energy balance	X				X						
Eat seasonally	X			X	X	X					
Focus on local food consumption			X	X				X			
Eat fish from sustainable sources						X			X	X	
Reduce food waste	X				X	X	X	X	X	X	
Eat a diverse diet				X						X	X
Choose foods with minimal or no packaging						X		X			

Nutritionists in the US have also had a long history of supporting such an approach. Over 30 years ago, Joan Gussow and Kate Clancy,^97^ members of the Society for Nutrition Education, argued for inclusion of sustainability considerations in formulating and educating consumers about dietary guidelines. Among other things, they were concerned about the limits of natural resources and the long-term stability of the food system. Ultimately, nutrition is not just about the nutritional health of consumers, they argued, it is also about the food system; we cannot have one without the other. Over a decade later, Gussow^98^ reinforced her earlier statements on sustainability and expanded them to include an emphasis on locally produced food and fair employment for workers in the food system. In 2007, the American Dietetic Association (now Academy of Nutrition and Dietetics) took a position, encouraging “environmentally responsible practices that conserve natural resources, minimize the quantity of waste generated, and support the ecological sustainability of the food system.”^99^

Given the mounting research on environmental damage and the links between diet and impacts on the environment, as well as the accepted practice around the world of considering sustainability in providing dietary guidance, the DGAC^1^ included sustainability in their recommendations for the US population in their scientific report published in January, 2015. Discussing the latest research on this in a chapter on food sustainability and food safety, they stated:

*consistent evidence indicates that, in general, a dietary pattern that is higher in plant-based foods, such as vegetables, fruits, whole grains, legumes, nuts, and seeds, and lower in animal based foods is more health promoting and is associated with lesser environmental impact ([greenhouse gas] emissions and energy, land, and water use) than is the current average U.S. diet.**^1^*

This is a straightforward, even modest reading of the overwhelming body of scientific evidence on both the relationships between diet and health and those between diet and the environment, which has now been reinforced with an updated systematic review by these experts.^100^ To allay concerns that the DGAC was recommending exclusion of any particular food or food group, they went on to say, “a diet that is more environmentally sustainable than the average U.S. diet can be achieved without excluding any food groups.”^1^

## A Question of Scope

The Secretaries of the USDA and Department of Health and Human Services decided that sustainability considerations would not be included in the DGA because of a matter of scope.^101^ For the scope of their mandate, they cited the 1990 National Nutrition Monitoring and Related Research Act as the provision of “nutritional and dietary information and guidelines … based on the preponderance of the scientific and medical knowledge.”^102^

However, the DGAC report did provide dietary guidance based on the preponderance of scientific and medical knowledge. Moreover, the original authorizing legislation indicated that there was nothing out of scope about including sustainability in the DGA.^102^

The National Nutrition Monitoring and Related Research Act has 3 titles: Title 1, Nutrition Monitoring and Related Research; Title 2, National Nutrition Monitoring Advisory Council; and Title 3, Dietary Guidance.^102^ Title 3, in a page and a half, calls for publication of the DGA every 5 years and is brief on the content of this report, stating simply that it should contain “nutritional and dietary information and guidelines for the general public.” Most of the rest of this Title is concerned with the federal government being consistent across agencies on the guidance that is distributed.

A review of the rest of the Act, to understand the context for Congress's approach to this topic, makes clear that the DGAC's inclusion of sustainability is within the scope of the authorizing legislation. The introductory definitions section makes it clear that nutrition monitoring and related research includes *food supply and demand determinations*. Title 2 on the Advisory Council, the presidentially established body that assisted in carrying out this Act, includes fields of expertise as selection criteria for Council members. One of the 3 sets of fields listed is food production and distribution, which includes a number of topics such as agriculture, and food-system management. Taken together, this makes it seem clear that congressional intent was to take a broad view of nutrition monitoring and dietary guidance that included food supply issues.

The 2015 DGAC were also consistent with previous developments of the DGA. The 2010 DGA had a Call to Action to ensure, among other things, that all Americans had access to nutritious foods and promoted a multipronged strategy that included a recommendation to

*develop and expand safe, effective, and sustainable agriculture and aquaculture practices to ensure availability of recommended amounts of healthy foods to all segments of the population.*^103^

Ensuring access to healthy food includes consideration of food safety, a topic that has been discussed in DGA reports for over a decade.^2^, ^103^^,^^104^

## Recommendations for Policy, Practice, and Research

Based on this evidence presented, the current authors make recommendations on dietary guidance policy, nutrition education practice, and research.

### Dietary Guidance Policy

Improving the nutritional health of a population is a long-term goal that requires ensuring the long-term health of the food system as well. Given the serious concerns about the natural environment, the ability to sustain expected increases in population with current dietary patterns, and the compelling nature of the science that link these issues, SNEB recommends that environmental sustainability considerations be included in future federal dietary guidance.

This position paper supports the original statement of the US DGAC that a diet with more plant-based foods than the average American diet would represent an improvement in both the environment and the health of the American consumer.^1^ Although there is little doubt about the veracity of this statement,^100^ it is general in nature. Future guidelines should not only consider environmental sustainability but provide more specific advice, such as substitution away from ruminant animal foods and toward other protein foods. This is partly so that consumers will have an easier time identifying specific actions they can take to improve their own health and reduce the impact on the environment. Although it is known that the bioavailability of some nutrients (eg, iron or zinc) from plant foods is lower than from animal foods,^105^ there are healthy vegetarian and vegan diets, which were reported in the 2015–2020 US DGA, along with a Mediterranean eating pattern. The benefits to the environment of such diets should be made explicit. The guidelines should also discuss reducing food waste as a benefit to environmental sustainability.

### Nutrition Education Practice

Dietary choices are a personal matter and there are many different dietary patterns that can lead to health. In discussing dietary recommendations, nutritionists can discuss both the health and the environmental impacts of food choices. For example, reducing animal foods, particularly ruminant animal consumption in the US, can have beneficial health and environmental effects, as can reducing overconsumption of food energy.^67^, ^81^^,^^106^, ^107^, ^108^, ^109^, ^110^, ^111^ Seafood from sustainable aquaculture^112^ can also provide a rich source of nutrients and reduce the impact on the environment, although feed sources for aquaculture production remain an important concern.^113^

Indeed, some individuals might find concerns about environmental impact more motivating than concerns about health when considering whether to make a dietary change. Based on the nationally representative American Climate Values Survey of 2014, >75% of Americans view clean air and water and unpolluted, toxin-free neighborhoods as rights that should be available to all people.^114^ The survey revealed that >70% of Americans are somewhat or very convinced that climate change is happening, and 25% claimed to be taking steps in their lives to keep climate change from getting worse. An additional 24% of the population worries about climate change but is unsure what to do about it. In other words, about half of Americans might be disposed to dietary advice that includes some mention of how food choice could affect the environment.

Such advice does not need to recommend absolutes. As the DGAC pointed out, a more environmentally sustainable diet can be achieved without excluding any food groups.^1^ Efforts to cut back on animal foods, such as Meatless Monday or the Protein Flip can reduce environmental impacts, improve health outcomes, and provide incremental solutions for consumers who desire them.^115^, ^116^ The Dutch have studied the frequency of meat consumption among individuals, even developing a lexicon around *flexitarianism*, a term for part-time meat eaters.^117^ Other suggestions, such as purchasing more organic products, can also have beneficial effects, although current research indicates that the benefits will not be as great as reducing consumption of animal foods. Reducing excess energy consumption and reducing food waste are also important suggestions that will have significant impacts on the environment.^59^^,^^109^, ^110^, ^111^

Expanding the knowledge of nutritionists in sustainability and environmental studies is needed to facilitate the types of dietary advice recommended earlier. Academic institutions can have a role in this by increasing such interdisciplinary topics in the training of future dietitians and nutritionists. Continuing education opportunities are also needed for current professionals. Such developments would benefit from collaboration with SNEB's Sustainable Food Systems Division.

### Research

Additional research on dietary patterns would assist in providing more concrete details for consumers about the specifics of a diet that is less impactful to the environment. Currently, the DGA publishes suggestions regarding several healthful eating patterns: a healthy US-style eating pattern, a healthy Mediterranean-style eating pattern, and a healthy vegetarian eating pattern.^2^ It would be desirable for future US dietary guidelines to include information about the environmental impacts of these patterns, as well as one that takes into account current consumption patterns in the US that are considered more sustainable. Although much is known about what makes up a sustainable diet in the aggregate,^118^ there have been relatively few studies on the diets of individuals who consume a more sustainable pattern, particularly in the US.^119^ This type of research could inform an environmental rating system of different diets, which would be useful to the large percentage of consumers concerned about this topic.

There are also significant gaps in the food LCA literature. In a recent review of 321 studies,^60^ there were no publically available studies on commonly eaten foods such as corn syrup, safflower and sesame oils, kidney or pinto beans, and most common herbs and spices. Moreover, only 12% of the entries in that review were conducted in North America. Although we know from other literature that GHGE associated with transportation and differences in production practices are dwarfed by the influence of diet composition, it would be helpful to have more precise figures on US production practices of commonly eaten foods. In addition, the vast majority of LCA studies focused on minimally processed food commodities; further development of widely consumed processed foods and likely substitutions such as plant-based meat alternatives will further improve diet-level estimates.

That review also pointed to another gap in the LCA literature: More information is needed on environmental impacts beyond GHGE.^60^ Many environmental concerns beyond GHGE, including biodiversity, water and land use, water quality, and air pollution, are far more regionally dependent, and US-based assessments are necessary to provide meaningful estimates of the influence of diet composition.

Finally, even the most comprehensive, evidence-based dietary guidelines will have only limited influence on consumers if they are not motivated to make changes and if these are not supported by other dissemination and behavior change efforts. Studies are needed to determine what motivates specific personality types to change their behaviors and whether strategies such as price changes, food labeling, messaging campaigns, etc, can promote both healthy and environmentally sustainable food choices.

## SUMMARY AND CONCLUSION

People want to know what to eat today, so it is incumbent on those of us who are knowledgeable about nutritional science and education techniques to provide the best advice based on the available evidence to date. Clearly this advice might change as nuances in the science are discovered and resolved. To the credit of those who formulated the initial legislation authorizing the DGA, they required publication of a report at least every 5 years for precisely this reason.

The best science we have today makes it clear that current environmental problems, including global climate change,^3^, ^7^ biodiversity loss,^10^, ^11^, ^12^, ^13^, ^14^, ^15^ land degradation,^16^ water shortages,^17^ and water pollution^18^, ^19^ demand urgent attention, threaten long-term food security, and are in part caused by current food choices and agricultural practices.^20^, ^21^^,^^24^, ^25^, ^26^^,^^32^, ^34^^,^^37^, ^38^, ^39^, ^40^, ^41^, ^42^ Addressing these problems while producing more food for the growing population will require changes to the food systems.^20^, ^21^^,^^24^, ^25^, ^26^^,^^32^, ^34^^,^^37^, ^38^, ^39^, ^40^, ^41^, ^42^ Dietary choices have a significant role in contributing to environmental impacts. These impacts could be lessened by choosing fewer overconsumed animal products, particularly food from ruminant animals, including more plant-based foods, and reducing excess calorie consumption and wasted food.^20^, ^32^^,^^37^, ^38^, ^39^, ^40^, ^41^, ^42^

Taken together, the science on environmental impacts of food choices and diet has several implications for policy, practice, and future research. First, federal dietary guidelines should include environmental sustainability considerations. Second, nutrition advisors and educators can convey both the health and environmental benefits of dietary choices. Finally, more research is needed on changing consumer behavior regarding sustainable diets and on various topics related to the LCA of foods, including studies on a wider variety of foods with a greater number of environmental impacts and on more foods produced in the US.
